# Topotecan and Cyclophosphamide in Adults with Relapsed Sarcoma

**DOI:** 10.1155/2012/749067

**Published:** 2012-07-18

**Authors:** P. Blanchette, D. Hogg, P. Ferguson, J. S. Wunder, C. Swallow, R. Gladdy, P. Chung, B. O'Sullivan, M. E. Blackstein, C. Catton, A. A. Gupta

**Affiliations:** ^1^Department of Internal Medicine, University of Toronto, Toronto, Canada; ^2^Department of Medical Oncology, Princess Margaret Hospital, University Health Network, University of Toronto, Canada; ^3^University Musculoskeletal Oncology Unit, Division of Orthopaedic Surgery, Mount Sinai Hospital, University of Toronto, Canada; ^4^Division of General Surgery, Mount Sinai Hospital, University of Toronto, Canada; ^5^Department of Radiation Oncology, Princess Margaret Hospital, University Health Network, University of Toronto, Canada

## Abstract

*Background.* The combination of topotecan and cyclophosphamide (TC) has activity in pediatric patients with recurrent sarcoma, especially Ewing's sarcoma (EWS). We sought to determine the toxicity of and response to TC in adults with recurrent sarcoma. *Patients and Methods.* Adults treated with TC from 2005 to 2010 were reviewed who received T = topotecan at 0.75 mg/m^2^/day (days 1–5) and C = cyclophosphamide at 250 mg/m^2^/day (days 1–5) every 21 days. *Results.* Fifteen patients, median age 31 years (range 17.5–56) had nonpleomorphic rhabdomyosarcoma (RMS, *n* = 6), EWS, *n* = 5, synovial sarcoma (SS, *n* = 2) leiomyosarcoma (LMS, *n* = 1), and desmoplastic small round cell tumour (DSRCT, *n* = 1). Median time to progression was 2.5 months (range 1.6–13.0). Partial responses were seen in 2/6 RMS and 1/2 SS. Stable disease was seen in 2/5 EWS, 1/2 SS and 1 DSRCT. The most common reason for stopping treatment was progressive disease 12/15, (80%). Hematologic toxicity was common; 7 (47%) patients required blood product transfusion, 5 (33%) patients had fever/neutropenia. At median follow-up time of 7.7 months, all but 1 patient had died of disease. *Conclusion:* TC combination is tolerable but has only modest activity in adults with recurrent sarcoma. Other regimens deserve exploration for this high-risk group of patients

## 1. Introduction

Approximately 13,000 patients are diagnosed with sarcoma annually in the United States, accounting for only 1% of all adult cancer diagnosis [1]. Certain sarcoma subtypes, including rhabdomyosarcoma (RMS) and Ewing sarcoma (EWS), are more common in children as well as in young adults, with adult patients having worse outcomes compared to their pediatric counterparts [1–3]. For example, almost 50% of adults with localized EWS are expected to relapse from their disease (reviewed in [4]). Even when treated with similar therapy, patients with EWS greater than age 18 relapse more frequently than younger patients [5]. Treatment strategies for relapsed sarcoma in adults are limited, and we evaluated if topotecan and cyclophosphamide chemotherapy was beneficial to this sarcoma population.

Cyclophosphamide (C) is an oxazaphosphorine alkylating agent causing 3′ nicks in DNA while topotecan (T) binds to and inhibits topoisomerase I when bound to free 3′ ends of DNA [6]. In combination, these two agents (TC) have demonstrated promising activity in children and adolescents with relapsed/refractory RMS with response rate of 67% [7]. Response to window TC therapy in newly diagnosed patients with metastatic RMS was 47% [8]. Similarly, response rate in pediatric patients with relapsed EWS was 32%–35% [7, 9]. Nonhematological toxicity was rare [7, 8]. In young patients with metastatic EWS, 57% had a partial response to TC window therapy [10]. Based on these results, TC has now been incorporated in a randomized study for patients with newly diagnosed localized EWS by The Children's Oncology Group (COG-AEWS07P1) [11]. 

The promising observations in pediatric patients and the limited nonhematological toxicity of this regimen make TC an attractive option for further clinical testing among adults with relapsed sarcoma. In the current study, we report the safety and efficacy of TC in adults with relapsed sarcoma. 

## 2. Methods

The medical records of consecutive patients with recurrent sarcoma treated with TC chemotherapy at Princess Margaret Hospital and Mount Sinai Hospital, Toronto from 2005–2010 were reviewed. Research ethics board approval was obtained for this study. 

All patients received topotecan 0.75 mg/m^2^ intravenously daily for 5 days plus cyclophosphamide 250 mg/m^2^ intravenously daily for 5 days, on a 21 day cycle. The use of granulocyte colony stimulating factor was variable. 

Radiologic response was rated according to RECIST [12]. Since this was not a formal clinical trial, systematic documentation of toxicity was not available. Need for transfusion and neutropenia associated fever was documented. Furthermore, any delay in chemotherapy and reasons for discontinuation of TC was documented. 

## 3. Results

### 3.1. Patient Demographics

Fifteen adults with relapsed sarcoma received TC chemotherapy as second or third line treatment. Patient characteristics are presented in Table 1. The median age of patients was 31 years (range 17.5–56). Patients had the following diseases: nonpleomorphic RMS, 6 (40%); EWS, 5 (33%); synovial sarcoma (SS), 2 (13%); leiomyosarcoma (LMS), 1 (7%); and desmoid small round cell tumour (DSRCT), 1 (7%). Median time to first relapse was 21 months (range 9–52). All patients had metastatic disease at time of TC treatment. 

### 3.2. Treatment

TC was used as 2nd line therapy for 12 patients and 3rd line therapy for 2 patients with RMS and 1 patient with SS (Table 2). A median of 4 cycles was delivered per patient (range 2 to 9). 

### 3.3. Disease Outcome

Response rate and time to progression are listed for all patients in Table 2. Overall response rate for RMS was 33%, EWS 0%, and other sarcoma 25%. Median time to progression was 2.5 months (range 1.5–13). TC was discontinued due to disease progression in 12 (80%), toxicity in 2 (13%), and patient refusal (not related to toxicity) in 1 (7%). At a median follow-up time of 7.7 months, 14/15 (93%) patients had died of disease progression. Failing treatment with TC, one patient subsequently had a complete response (CR) to an IGF-1R antibody and remains in CR at 28 months.

### 3.4. Toxicity

Hematological toxicity was observed in 7 (47%) patients (Table 3). Seven patients (47%) required at least one blood transfusion and three patients (20%) required at least one platelet transfusion. Five patients (33%) had at least one episode of febrile neutropenia. 

## 4. Discussion

This is the first paper reporting outcomes of adults with relapsed sarcoma who received salvage chemotherapy with topotecan plus cyclophosphamide. Although the combination seems tolerable, there is less activity observed in adults with relapsed EWS and RMS compared to previously reported pediatric series [7, 9]. There were no responses seen in the 5 adults with EWS.

Due to the rarity of “chemo-sensitive” sarcomas in adult oncology practices, it is difficult to design trials to systematically evaluate regimens for patients with relapsed EWS or RMS. In this regard, extrapolation from pediatric series is common; however, our data, though limited in sample size, caution against assuming equivalent outcomes across ages. There are likely differences in primary therapy delivered between patients included in this series and the previously published pediatric patients. For example, pediatric RMS patients would have likely received vincristine/actinomycin/cyclophosphamide-based chemotherapy, rather than that containing doxorubicin as in our series. Nonetheless, the 2 patients with RMS who attained a partial response to TC chemotherapy had previously received vincristine/doxorubicin/cyclophosphamide alternating with ifosfamide and etoposide. Response to TC does not seem to correlate with prior exposure to alkylating agents [7]. TC was used as 3rd line therapy in 2 others with RMS who did not respond. In EWS, older patients more commonly present with large pelvic disease [13], and are offered local control later compared to that prescribed by pediatric protocols [3] perhaps contributing to differences in response to salvage therapies. 

There is substantial interpatient variability in the pharmacokinetics of topotecan with 10-fold variability in systemic clearance of the drug [14]; however, to date there have been no studies examining pharmacokinetic targeting in patients with EWS or RMS. Cyclophosphamide pharmacokinetics are also quite variable, and age-related differences in drug handling between pediatric and adult patients may, in part, play a role in outcome of patients with sarcoma [15–19]. The haematological toxicity in this study was modest and comparable to prior studies in which there was routine use of growth factor support [7, 9].

In conclusion, topotecan plus cyclophosphamide combination is tolerable but has less activity in adults with recurrent sarcoma than previously reported in pediatric series. It may have more benefit when used as 2nd line, and its use as 3rd-line therapy should be limited. Confirmation of our preliminary data is required with larger adult series; however, patients with relapsed EWS should perhaps be offered alternative therapeutic opportunities such as temozolomide plus irinotecan [20, 21] or clinical trials exploiting novel targets. 

## Figures and Tables

**Table 1:** Patient characteristics.

Patient characteristics	Number (*N*)
Total	15
Diagnosis:	
Rhabdomyosarcoma	6
Ewing's sarcoma	5
Synovial sarcoma	2
Leiomyosarcoma	1
DSRCT	1
Gender:	
Male	8
Female	7
Age:	
Age at diagnosis (years)	mean: 30, (range: 16–56)
Age at relapse (years)	mean: 31, (range: 19–57)
Topotecan/cyclophosphamide	
2nd line therapy	12
3rd line therapy	3
Time to 1st relapse (months)	mean: 21, (range: 9–52)

**Table 2:** Treatment and disease outcome in patients treated with topotecan/cyclophosphamide.

Patient	Age at TC treatment	Diagnosis	Time to relapse (months)	Prior therapy	TTP with TC	Best response with TC	Outcome
1	51.5	RMS	11.8	VDC	1.6	PD	DOD
2	26.3	RMS	24.8	VAC, then VDC	2.0	PD	DOD
3	31.0	RMS	15	VAC, then VDC	2.3	PD	DOD
4	29.5	RMS	47.6	VDC/IE	4.3	PR	DOD
5	23.3	RMS	16	VDC/IE	1.8^∗^	PR	DOD
6	49.6	RMS	19	VDC/IE	2.5^∗^	PD	DOD
7	19.0	EWS	38	VDC/IE	1.5	PD	ANED
8	43.2	EWS	35.4	VDC/IE	5.3	PD	DOD
9	23.3	EWS	14.3	VDC/IE	2.3	PD	DOD
10	17.7	EWS	13.1	VDC/IE	6.1	SD	DOD
11	56.6	EWS	13.0	VDC/IE	13^∗^	SD	DOD
12	32.0	DSRCT	10.1	VDC/IE	7.0	SD	DOD
13	46.7	SS	14.4	Dox, then Gem	5.6	PR	DOD
14	24.8	SS	42.5	AI	5.6	SD	DOD
15	57.4	LMS	80.5	AI	1.8	PD	DOD

TTP, time to progression; RMS, rhabdomyosarcoma; EWS, Ewing's sarcoma; DSRCT, desmoplastic small round cell tumour; PD, progressive disease; PR, partial response; SD, stable disease; DOD, dead of disease; ANED, alive no evidence of disease.

^∗^Discontinued TC for other than disease progression.

**Table 3:** Toxicity related to topotecan/cyclophosphamide chemotherapy.

Toxicity	Number (*N*)	Percentage (%)
Febrile neutropenia	5	33%
Patient requiring transfusion	7	47%
Blood	7	47%
Platelets	3	20%
Discontinuation of therapy	2	13%
